# Enstranglements: Performing Within, and Exiting From, the Arts-in-Health “Setting”

**DOI:** 10.3389/fpsyg.2021.732957

**Published:** 2022-01-05

**Authors:** Frances Williams, Becky Shaw, Anthony Schrag

**Affiliations:** ^1^The Faculty of Arts, Science and Technology (FAST), Wrexham Glyndwr University, Wrexham, United Kingdom; ^2^Department of Art and Design, Sheffield Hallam University, Sheffield, United Kingdom; ^3^School of Arts, Social Sciences and Management, Queen Margaret University, Musselburgh, United Kingdom

**Keywords:** performance, space, institutions, critique, Arts in Health, university, hospital

## Abstract

The following text explores performative art works commissioned within a specific “arts and health” cultural setting, namely that of a medical school within a British university. It examines the degree to which the professional autonomy of the artists (and curator) was “instrumentalized” and diminished as a result of having to fit into normative frames set by institutional agendas (in this case, that of “the neoliberal university”). We ask to what extent do such “entanglements,” feel more like “enstranglements,” suffocating the artist’s capacity to envision the world afresh or any differently? What kinds of pressures allow for certain kinds of “evidence” to be read and made visible, (and not others)? *Are You Feeling Better?* was a 2016 programme curated by Frances Williams, challenging simplistic expectations that the arts hold any automatic power of their own to make “things better” in healthcare. It included two performative projects – The Secret Society of Imperfect Nurses, by Anthony Schrag with student nurses at Kings College London, and Hiding in Plain Sight by Becky Shaw (plus film with Rose Butler) with doctoral researchers in nursing and midwifery. These projects were situated in a climate of United Kingdom National Health Service cuts and austerity measures where the advancement of social prescribing looks dangerously like the government abnegating responsibility and offering art as amelioration. The text therefore examines the critical “stage” on which these arts-health projects were performed and the extent to which critical reflection is welcomed within institutional contexts, how learning is framed, expressed aesthetically, as well as understood as art practice (as much as “education” or “learning”). It further examines how artistic projects might offer sites of resistance, rejection and mechanisms of support against constricting institutional norms and practices that seek to instrumentalise artistic works to their own ends.

## Introduction

The room looks sterile. Its floors and walls are a bland colour. There is a pile of nondescript stackable chairs in a corner. A pleated curtain draws around an empty bed. There is a trolley with some medical equipment on top of it. There is a table with a computer and a box of latex gloves. It appears to be a perfectly normal hospital room, and everything is still and quiet, like a stage before the actors arrive.

There is something wrong with the cupboard space, however. Some pillows appear to have fallen out. These attract a second glance and on closer inspection, a dark shape appears that seems out of place. Looking closer we see a shoe, a leg, and then it becomes apparent it is a person – a hospital worker – hiding, waiting. Someone enters the room, giggling, looking behind the curtains and behind the chairs. The person in the cupboard tries to hold their breath, become as small as possible and to disappear into the environment. But they are soon found, and the seeker helps the hider unfold herself, all the while both laughing. They disappear into another booth, looking for more people hiding.

The performative work, Becky Shaw’s Hiding in Plain Sight is discussed in greater depth below, but is a useful place to begin, as it poses a neglected question about how or where arts and health practices collide or coalesce, and the types of “space” in which such actions take place.

In an academic paper that takes stock of recurring challenges besetting research in the field of Arts in Health, a number of gaps or “lacunae for further investigation” are usefully identified (Raw et al., 2012). These authors call for future studies that can help theorise the “nature of the project space created by artists” and those “participatory artists working within health and community settings” in particular. Some of the affective qualities already ascribed to such spaces, created by artists, are listed and comprise: “a sanctuary or suspended, protected space, where new things are possible” (White, 2004; Gould, 2005; Sixsmith and Kagan, 2005; Kilroy et al., 2007; Putland, 2008). Spatial concepts drawn from psychoanalytic traditions – such as platforming and liminal space – are also referenced in order to show how change is made possible within creative contexts, spaces and structures (Atkinson and Robson, 2012). Load-bearing metaphors have also been deployed – alongside spatial ones – to show how art practice offers “a means of support to carry one over the threshold of change,” (Elliott, 2011).

Our paper aims to do a couple of things. Firstly, to respond to the call made above; unpacking, complicating and extending the challenge to explore the “space” produced by arts practice in healthcare. We explore two live artworks that made space “for” performance – “through” performance – within the pre-existing structure of an institution of higher education. The projects were developed between artists and healthcare students around the theme of Utopia. Yet, in their treatment and interpretation of this topic, they resist the idea of any exo-space or ideal society located in some far away place. Instead, they situate potentials for change within “secret,” “hidden,” and “fugitive” spaces close-at-hand within the institution.

Secondly, we wish to bring into relation with “Arts in Health” research, those discourses and practices which draw on the long traditions of “institutional critique” in the arts. This tradition offers a context in which to discuss the terms on which “criticality” in arts-health is drawn, offering relevant points on the nature of artist agency relative to institutional power – including supra-national institutions such as the World Health Organisation – alongside those more everyday institutions we more commonly operate with and within (the local-global context of the University, or Contemporary Arts Institution).

In the following, we explore the conditions of the commissions and how the particular constraints, contradictions and affordances of the (concurrently, laden and dissolute) institution, germinated these respective live works. Exploring ideas of utopia, (Jacoby, 2005), we explore these works as examples of “instituent practices” (Raunig and Ray, 2009). Like “third wave” (the third generation of) forms of institutional critique explored by Raunig and Ray, the commissions here were utterly responsive to, and dependent upon, the conditions that generated them. Yet these same conditions also produced the projects’ tendency toward becoming invisible. Shaw and another artist – Anthony Schrag – “suffer” this institutional evaporation at the same time as working with it tactically.

We conclude by exploring the extent to which this research into the “spaces” created through Arts in Health practice can contribute to a different kind of research “agenda.” Rather than seeking to clarify, simplify or extrapolate, we aim to capture a “whole” about the reality of the commissioning context here. Though no less “evidence,” such an approach refuses debate about quality or efficacy to propose a value in making a critical space for others. Importantly, we argue, it might offer an alternative way to do “criticism.”

## The (Heavily Loaded) Commission

Devised by artists Becky Shaw and Anthony Schrag, the two projects, *Hiding in Plain Sight* and *The Secret Society for Imperfect Nurses*, were part of an education programme for healthcare students developed at King’s College London (KCL), curated by Frances Williams^1^. *Are You Feeling Better?* was the title of a programme very deliberately built around concepts of human potential and “betterment.”

Williams’ intention was to deploy the phrase playfully, if not to undermine, then certainly to throw open, any simplistic prescription of culture as an automatic good, something to be consumed in order to cure ills. The programme was devised, instead, as a way to hold the promises of healthcare to account and also question the goals of academic achievement set in place by higher education. Any sense of ambivalence the question might have been able to foster here was forged within, as much as against, the wider institutional agenda – on the *a priori* terms set out for such (self) reflection and critique.

*Are You Feeling Better?* was just a single strand in a far broader, expansive season of events that ran across KCL’s many schools and departments in 2016. It represented the healthcare-student-education component of a year-long celebration of Thomas More’s book, Utopia, and was described as the “largest ever” festival of its kind. The 500th year anniversary of the book’s publication was marked by an array of prestigious cultural bodies across the United Kingdom capital, involving in turn, many high-profile contemporary artists (such as Jeremy Deller).

King’s College London (KCL) is London’s oldest and largest education and research establishment. Its own mission statement harbours no small degree of Utopian intent: “through world-leading and outward-looking research, focussed on meeting societal need, King’s will make the world a better place” (Sholette, 2010, 2015; Utopia 2016 website, 2016). KCL chose to align their own strategic mission alongside the promise of More’s book, branding 2016 “a year of imagination and possibility.”

*Throughout UTOPIA 2016… people from all walks of life will be invited to experiment with new ways we might live, make, work, play and dream. We will create physical and virtual spaces where positive visions are nurtured, supported and celebrated, and where anything is possible (Utopia 2016 website, 2016).*

This marketing strapline was typical of the high ambitions KCL claimed for itself. It had, at this time, a prominent cultural leader in Baroness Deborah Bull (2012 – 2019) who set in motion various collaborations across KCL’s inter-disciplinary territories, as well as fostering partnerships between KCL and neighbouring cultural bodies. These included Somerset House (an arts body who now occupy the grand 17th century buildings of the former tax office, alongside The Thames) as well as The Courtauld Institute of Art (keeper of historic collections of priceless works of art). As well as developing key strategic partnerships between these eminent organisations, Baroness Bull also created a brand new organisation, namely the Cultural Institute.

The Cultural Institute was intended to act as an internal catalyst for change at KCL, one that could work to help respective departments to collaborate, enabling them to “connect through culture” (Utopia 2016 website, 2016). An independent, freelance Producer (Andy Franzkowiak) was engaged by the Cultural Institute to explore the theme of Utopia through a summer exhibition at Utopia 2016 website (2016). Like Williams, he also enlisted artists, researchers and students around this theme. But this programme was more squarely titled, *Paths to Utopia*, and focussed on collaborations with science staff, rather than having a particular focus on health or education *per se.*

It was intended that artists from *Are You Feeling Better?* would contribute, in smaller part, to this exhibition. They were indeed included as part of a rotating programme held in an adjunct space, titled *Utopia Lab*. The materials displayed here from *Are You Feeling Better?* including films and a booklet, were presented as documentation (as they unfolded out of sight, mainly as interactions between people in preceding months behind the scenes of this public facing exhibition). In this respect, the power-configuration was traditionally orthodox: arts education projects were situated in a shady demimonde and accorded lower status than the more spectacular forms of fine art which are more traditionally respected.

*Are you Feeling Better?* was thus held within many concentric circles of devolved commissioning (and similarly smaller allocations of financial resource). These configurations already differentiated what was “good” from what was “better,” what was public-facing and what was hidden, and what spatial and affective perimeters the works were supposed to obligingly perform within. Part of Utopia Lab’s planned limitation was informed by inherited assumptions about curation and its relation to education that have been articulated by those within the field. “Gallery education is typically situated at the edge” within institutional formations and is “overshadowed” by other activities (Allen, 2008, 9). While the Utopia Lab space alluded to the fluid, dialogic, intentions that lay behind New Institutionalism, the respective remits in this case were split across two rather than one curator role. In this way, they remained separate and fixed (and unequal).

New Institutionalism, so named, (Farquharson, 2013) was built on the affordance of independent curators who, in the increasingly flexible working terrain of the 1990s, brought a desire to establish new power relations and commissioning configurations within the (art) institution. They embraced education’s dialogic potentials (if not the sub-field of “gallery education” *per se*) (Mörsch, 2011). Key curators became embedded in, or built, small and medium-sized arts organisations at this time, intent on working for change “from within.” In the words of one such curator, these smaller-scale models were “proud to be maladjusted” as they did “not adjust themselves to an art community obsessed with knowledge, power, and scale.” (Huberman, 2011). While the rhetoric of the “experimental” *Utopia Lab* nodded to the motifs of new institutionalism, it was encompassed and nested within the broader Utopia programme – one which measured its impacts though size and scale.

Williams was engaged, then, as a subsidiary freelance producer to explore how healthcare students could be enticed from KCL’s four Health Faculties (Nursing and Midwifery, Medicine, Dentistry and Psychology, Psychology and Neuroscience) to engage with artists around the theme of “health utopias.” She reported to the Research and Education Manager at the Cultural Institute who in turn reported to the Director, thus positioned at the end of a line of complex managerial structures, not employed as part of the institution, but a participant in the gig-economy which operated at its fringy edges. A Student Engagement Manager who directly facilitated Williams’ personal introductions to staff and students left toward the end of the project. Working on a similar contractual basis to Williams, this was a vital human link, (if one easily cut).

Passing down-the-line any sense of potential within this distinctly hierarchical arrangement of finance, responsibility and power, the space in which Shaw and Schrag could develop their work was already informed by a series of prior intentions and power-relations (and sly, counter intentions too, one might conclude). The complex and contradictory terms of the commission thus exerted great influence on how their respective live works could be made, who they might engage with, and how the work would be subsequently received.

## Backdrop to the “Action”

On inheriting this brief, the possibilities open to the curator and the artists felt as constrictive as they did expansive. “My task of engaging hard-pressed students to give time to projects whose outcomes no-one could yet imagine, felt challenging, to say the least” (Williams, 2016). Space for students to work with artists only became apparent “in the fissures and cracks between study and work placements” (Williams, 2016).

Students were not only pressured by exam expectations but were also working at a time of a high-profile dispute between NHS staff and its employees. The Junior Doctors strike provided a backdrop of anger centred around the struggle to maintain existing contractual terms amongst medics who had recently graduated. Students staged walkouts in support and suspended regular work patterns in order to protest and protect their future pay levels and contractual working conditions.

This dispute had been characterised by the Health Secretary and his collaborators, as one in which the designation of weekends as rest time had become unworkable, presented as an unaffordable utopian ideal. As one nursing student, who later became a project participant noted: “In a world of underfunding, understaffing, excessive workloads and crushed ideals, the notion of utopia seemed fanciful” (Jackson, 2016).

Acknowledging these feelings of disillusionment, Williams hung-out on picket lines and saw the need to make any “cultural offer feel a little bit counter-cultural too” (Williams, 2016). Warned away from engaging too directly in political developments through the commissions, she was steered by her commissioners, toward enlisting healthcare student populations previously unreached by previous engagement projects. As a means to this end, Williams was introduced to two vocal student community leaders, Mavis Machiori, (a Ph.D student in nursing and midwifery) and Tim Owen Jones, (a student representative for nursing students). Williams presented the project offer to them as an unusual opportunity for healthcare students to develop a “special kind of space” together with invited artists, “to talk about work, but not as part of work” (Williams, 2016).

Within this parallel trammel, it was hoped that some degree of critical distance and reflection might be enabled through the material processes of working with artists. A similar possibility was captured by the journal About Performance when seeking to foster a special edition- they noted a desire to create “the necessary distance for health professionals and health consumers to become critically reflexive - to see more clearly what values and identities are (re)produced by the performativity of health systems – and to intervene in processes of systemic change.” (Call for Papers for Performing Care, About Performance 2018 journal now ceased).

But in an educational and institutional environment where high performance is drilled into the student ethos as a prerequisite for success and resolution, and in a clinical environment where evidence-based care is so firmly entrenched, what space is there for a “slacker,” more open-ended enquiry? The project began to explore how to counter this multiple context of suffocating pressures, and experiment with how projects could nudge or tilt these paradigms in a performative, process-led manner. Williams, Shaw and Schrag were also cognisant that the institution’s emphasis on the final exhibition would hierarchically demonstrate knowledge, value and learning rather than generating any reflective space to examine the student’s own individual contexts, politics and learning. In this sense the projects did not separate out the usually delineated contexts of art, healthcare and culture (or to take for granted the construction of “art and health” as a separate world to “art”) but attempted to grasp and work with their combined pressures.

## The Secret Society for Imperfect Nurses

The Secret Society of Imperfect Nurses emerged out of conversations between Anthony Schrag and student nurse, Tim Owen Jones. It sought to provide a space to discuss the pressures of perfection – the utopian values that student nurses felt they had to always live up to. As well as being based around the question of whether imperfect people could embody utopian values, more importantly perhaps, it also asked questions about whose utopian values nurses might be expected to embody.

**FIGURE 1:**
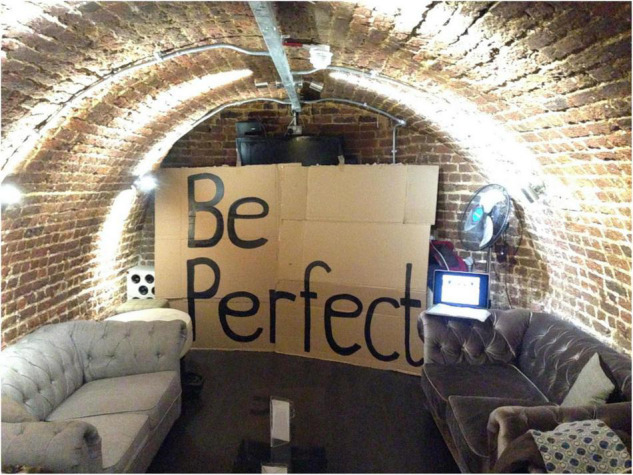
Image of meeting space from the Secret Society of Imperfect Nurses. Anthony Schrag.

Conversations focussed on how pressures of perfection are both professionally and socially demanded; the internal drive to adhere to those high standards are premised upon emotional labour and historical assumptions of nursing practice. To be a nurse is to live in the shadow of Florence Nightingale; to be endlessly hardworking; to have inhuman levels of empathy; and to never, ever make a single mistake: to be utopian.

In *Picture Imperfect: Utopian thought for an Anti-Utopian Age* (2005), Russell Jacoby argues that utopias are important because it is through them that we develop aspirations, and from those aspirations we develop political change:

*Utopian thinking does not undermine or discount real reforms. Indeed, it is almost the opposite: practical reforms depend on utopian dreaming….Utopian thought consists of more than daydreams and doodles. It emerges out of and returns to contemporary political realities….this contradiction defines the utopian project: it partakes at once of the limited choices of the day and unlimited possibilities of the morrow (Jacoby, 2005:146).*

Utopias therefore have a political imperative because they espouse the promise of an alternative world order. However, due to the heterogeneous and pluralistic nature of societies that contain different and often oppositional politics, the plurality of these political agendas and the impossibility of each and every utopian future being true means that utopias are always bound to fail. Not all utopias can come to fruition, and it is those with the most resources who will make their perfect world more true and real than those less resources. Utopias are therefore built by, and for, the powerful.

Jacoby does not argue that, because of this, we should consequently abandon the search for a better world, but rather he suggests that imaginary possibilities of utopias (in general) are an important social and developmental mechanisms and not mechanisms of policy and legislation. He recognises that utopias have a generative relationship to conflict, and that they are useful but only if we accept that they are all bound to fail.

What, then, of the notion of the Utopian Nurse? How do their utopian failures become acceptable? How can nurses perform imperfection? In current political climate of the United Kingdom, the performance of the perfect nurse is one that subsumes his/herself to the will of the institutional agendas that do not serve them. How can this be resisted? Can the performance of imperfection offer some kind of resistance to the forces of measurement and accountability that demand certain forms of perfection?

Out of the discussion of the possibility of the Utopian nurse, *The Secret Society of Imperfect Nurses* was instigated. The work offered a structure that arrived from a lineage of proto-organisations, or “mockstitutions” (Sholette, 2010, 2015), but also held the potential to become a real organisation. The work made a space to explore the expectations and limitations of being a professional carer in today’s NHS, as well as a wider reflection about the notion of the perceived utopia of the Healthcare system, in general.

Complex temporalities were at work in how the industrial dispute was being fought and engineered through various avenues of public discourse. The NHS was established, one academic argues, “as a utopian enclave prefiguring an idealised non-capitalist future” (Harrington, 2009). Market forces were/are threatening to penetrate this once protected space, an activist nurse contends on twitter: “The NHS nursing workforce crisis will be solved by investment, not by looking backwards with rose tinted nostalgia” (Tiplady, 2019).

*The Society* met in a basement bar, (a private members drinking club for medics) repurposing a corner and constructing a new function from it. Anonymous cards were used to draw in members. The creation of fake names created a “secret” atmosphere that utilised aesthetics of underground, resistance movements not usually associated with healthcare. It was a clandestine space to admit one’s flaws, to critique others, and to analyse the systems that demanded the impossible. The work offered a way to perform that was counter to the expected role of a nurse. It was a movement against perfection.

The aesthetic conceit of resistance was aligned with Chantal Mouffe’s notion of agonism in that it was an artistic space that did not attempt to totally reject the ideas of the perfect nurse, but rather provide a space to explore what that notion of what that idea means. Mouffe writes:

*Those [artists] who advocate the creation of agonistic public spaces where the objective is to unveil everything that is repressed by the dominant consensus are going to envisage a relation between artistic practices and their public in a very different way than those whose objective is the creation of consensus – even if that consensus is considered critical consensus. According to the agonistic approach, critical art is art that forms a dissensus – that makes visible what the dominant consensus tends to obscure and obliterate, aiming to give voice within the existing hegemony (Mouffe, 2007).*

The intention of an agonist intervention within the public space is not to make a total break with the existing order and suggest an alternative political utopia, but to subvert that order, and provide new subjectivities. In other words, it is art’s role to provide a “potential for transformation,” rather than be a political act that guides the transformation itself. *The Secret Society* was therefore a productive space for student nurses to find points of contact and resistance and did not aim to fix perfection and replace it with another ideal, but rather provide a moment of resistance from which new potentialities could develop. As an artistic space, rather than a pedagogical or political group, the imagining of other possibilities provided gaps within the armour of perfection (as well as the assumptions of imperfection as “failure”). As has been suggested: “art is a wonderful place where you can reflect on the failures(s) of utopia” (Bishop and Groys, 2009).

Hailing from the tradition of institutional critique, Gerald Raunig’s term “instituting” offers a useful institutionally inflected form of agonism. He describes “a site of productive tension between a new articulation of critique and the attempt to arrive at a notion of ‘instituting’ after traditional notions of institutions have begun to break down” (Raunig and Ray, 2009). He describes practices that are still geared toward critique but offer an actualisation of a future, “a process and concatenation of instituent events.” This exceeds mere opposition to institutions: it is not leaving the institution but “fleeing” institutionalisation. Raunig and Ray (2009) suggests that the “specific competencies of art can be deployed to spur on a general reflection on the problems of institutions, the predicaments of critique and the openings for new ‘instituent’ practices.”

Raunig and Ray revisit the forms of artistic institutional critique from the 70s and 80s and note that the 80s practitioners (Andrea Fraser, in particular) articulated a conviction that it was impossible to function, be legible or effective outside of the art institution. By contrast they see contemporary “figures of flight, of dropping out, of betrayal, of desertion, of exodus” (Raunig and Ray, 2009), as a refusal of cynical invocation of hopelessness (such as “there is no alternative” first asserted in the 1980s by the Thatcher government). Here, Schrag’s coming together in the name of imperfection offers a similar kind of mechanism; it maintains a commitment to organising together, but around a different set of values.

To return to the challenge then, outlined at the beginning of this paper, it is useful to think about both the time and space of *The Secret Society of Imperfect Nurses*, in relation to Raw et al.’s interest in the spaces created by arts-in-health as “sanctuary or suspended space” (Raw et al., 2012). It would be absurd to consider Schrag’s *Society* as a sanctuary, a term as pious as the perfect nurse the society worked to debunk. However, the space of the *Imperfect Nurses* (and the performances therein) does mark a type of temporal suspension that exists in and without the institution.

Once Schrag was no longer leading the *Secret Society*, we assume it ceased to exist, but we don’t know. Possibly one “successful” outcome of the project might have been that the institution attempted to “institute” it, recognising that, ironically, the process might make the students become better nurses (luckily this didn’t happen!) It is also possible that the group continues, led by student nurses. While on one hand, this might reflect a genuine agency for the project, this would equally make it easily co-opted into institutional narratives of successful social engagement and impact.

Maybe the *Society’s* transformation of space and thought continues through here-say and myth, or maybe it has vanished into the ether. We will never know where or when the para-world that the work constructed begins and ends. While there was some pressure to evidence or account for the healthcare student’s engagement, Schrag made no aspect of the society visible for public exhibition. Partly this was because to make it visible would have undermined its secretive and mythic status, and also because these very performances of visibility and accountability were part of what Schrag and the society were working to disavow. Agonistic re-imagining is not intended to be productive to the institutions it critiques. Instead, it is intended to “makes visible what the dominant consensus tends to obscure and obliterate” (Mouffe, 2007).

## Hiding in Plain Sight

Becky Shaw was invited to work with a group of healthcare practitioners who were undertaking doctorates. They described doing a doctorate as a tactic to change their status, enabling their work to be legible as knowledge, so they had greater agency and influence to change a system they knew, inside out, as practitioners. The problem of their transitional, uncertain identity and how they belonged in institutional space became the starting point for the work.

**FIGURE 2:**
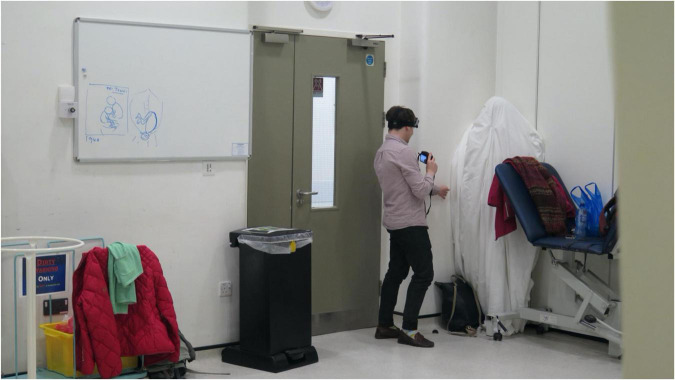
Image of doctoral students playing hide and seek in Hiding in Plain Sight, Becky Shaw.

This group of students were articulate and aware; doubtful, cynical and curious about the value of any art process. They were uncertain whether this was education, community engagement or art and questioned who the project might serve. Rather than seek to clarify or resolve this tension, in the name of comfort or keeping the group engaged, Shaw worked to enable the group to explore this. The conversation began at Florence Nightingale Museum, by talking about the spatial transition from practitioner to researcher. Nurse Matt Alder likened his “in-between” position to the precise definition of utopia, a “no-place.” The group talked about how, as healthcare practitioners, their time, role and mode of occupying space was fixed, but doing a doctorate produces an uneasy structure and a discomfort about what it means to “look busy” in un-programmed time. This entailed them having to learn how to occupy different spaces and having uncertain affiliations to their community. Many of them continued to work in their professional roles, either as research contexts or to maintain professional skills. The double role caused great anxiety and pressure.

Shaw noticed how many times forms of visibility – seeing and appearing – haunted conversations with the group. The group talked about the powerful and unravelling effect of witnessing death and trauma, alongside scientific and legalistic regimes of observation, such as the midwifery requirement to keep birth records for twenty-one years. They also talked about the reality of patient-staff and researcher-subject relationships as contingent, intimate, blended, often not adhering to the simplistic managed discrete separation deemed “professional.”

Williams negotiated access to a Simulation and Interactive Learning (SaIL) Centre – a mocked-up ward environment that could be booked by staff. Like the *Are you Feeling Better?* commission, the SaIL Centre squeezed together education, learning and healthcare practice in one space. The group were encouraged to explore the material nature of the space and their relationship to it- a space that they never get to attend to or “see”: when using it students must “believe” it is a real ward, rather than attending to its fictional status. Shaw invited the group to use bridge cameras as instruments to “look” with. They looked under and over furniture, closely at the weave of blankets, they looked at the patients’ angle of view from the bed and they unpacked and laid out the emergency crash kit – a routinely repeated, fundamental part of critical care training. The well-worn ideal of moving research from “bench-side to bedside” (a mantra about bringing research straight from the lab to the patient) became an ironic joke as furniture was literally moved around. The experience of engaging with the material of the ward – and the conversations about visibility – coalesced into a decision to play hide and seek: a kind of material experiment with appearing and disappearing. An awareness of the way participants are represented photographically in social practice (the smiling, successful group), education (the successful achievers) and research projects (the research subject) generated a refusal to simply represent the gameplay.

Instead, representation and photography were understood as part of the logic of the game. The Seeker had to seek with a digital camera, their goal to catch an image of the Hider, while the Hider wore sound recording equipment to “catch” their silence. The 2 hrs of play involved furniture sliding across the room, gasping bodies trying to hold breath, bodies falling out of cramped positions and explosive laughter of discovery. A plastic patient dummy lying in bed heaved with laughter as a Seeker crept nearer the Hider, hidden underneath the dummy. A Hider wrapped up in hospital cellular blankets withdrew deeper into the ward curtains, like a snail, as the Seeker’s outstretched hand clutched at something that seemed part hair, curtain and blanket. The footage contains strange round dark forms, not recognisable as bodies, twitching blankets, and askance angles down sides of beds. Rhythms of suppression and constraint and eruption and outburst marked the physical encounter.

After the gameplay, the group were invited to read Walter Benjamin’s text, *A Child Hiding* (Benjamin, 1928) together, as a tool to think about the game in relationship to their working lives. Moving round the space, the group noted that the visible relationship between skin, bodies and the material of the ward started to be much less distinct and all of it became a kind of animate skin. Benjamin describes this as being enclosed in matter or even becoming part of matter – “behind door he is himself door” (Benjamin, 1928).

Benjamin writes about how being found can “petrify” the Hider, weaving him “forever as a ghost in the curtain,” banished for life “into the heavy door” (Benjamin, 1928: 74). The group reflected on the possibility that on one hand hiding might mean that they were forever fused and petrified into the institution, or it might offer an escape from the “performance” of the institution. They saw this possibility as a desirable state of reverie or an escape, an exit from the pressure of performance. Like Schrag’s *Secret Society* the Hiders are embedded in the institution, but there is also a sense of a line of flight, an exit, from a particular form of the institution and the institutional.

The group also reflected on what was left after the hiding game has taken place. Together they read the part of the text where Benjamin talks about the spaces left after children collect Easter eggs and likened it to the impact of their own hiding, saying, “It’s like a body shape has been left in the place. By hiding in this space you have made a black hole, a new negative space” (Participant, *Hiding in Plain Sight* 2016). This phrase was unexpected and peculiar, suggesting that they had exited but also that they leave a type of dark matter. This drew verbal connections with Sholette’s (2010) “dark matter” metaphor for invisible labour that supports the construction of other people’s more visible roles. The parallels with Schrag’s *Secret Society* are also apparent: the perfect nurse is an unending, invisible service for others. Schrag’s *Imperfect Nurses* lurk in dark spaces, the *Secret Society* making visible a disavowal of visibility.

**FIGURE 3:**
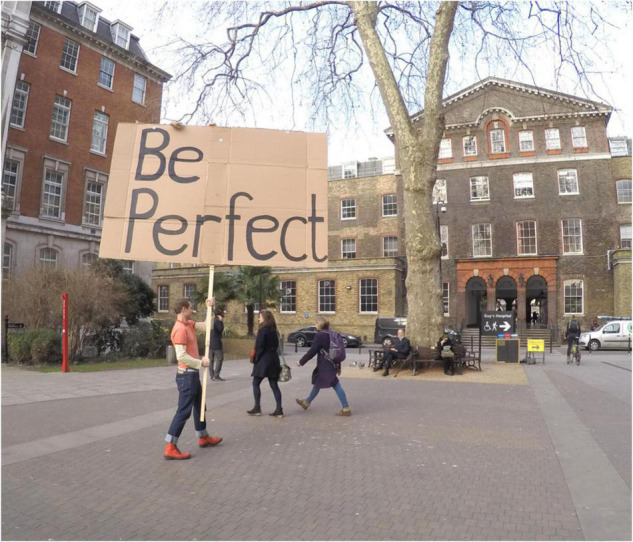
Schrag holding “be perfect” placard from the Secret Society of Imperfect Nurses. Anthony Schrag.

The use of the term “black hole” also enabled reflection on the effect of temporary, speculative works (or maybe all works) after they have gone. Like Schrag’s *Secret Society*, Shaw’s *Hiding*, leaves material and structures unchanged but leaves an affect, or charge, that might change how the space feels afterward for those who made it. These are the kinds of “impacts” that rarely register in institutional contexts, though they may be more profound and affecting than any metric or body count. Too often, assessments are burdened by the desire to grow an audience separate to the shared process of making the event happen between those actors already present, alive to what they can make happen together.

**FIGURE 4:**
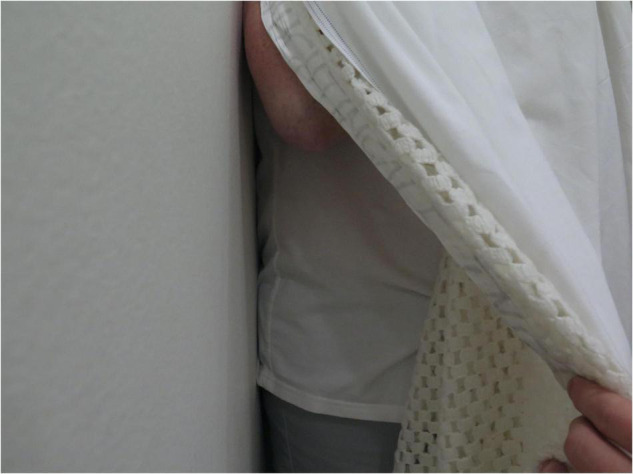
“Seeker” trying to grab “Hider” during hide and seek game, from Hiding in Plain Sight, Becky Shaw.

## The Booklet: Outcomes as Evidence

In the subsequent booklet Williams developed and edited for inclusion in the *Paths to Utopia* exhibition, she proposes that the projects took place in a “fugitive space” (Williams, 2016). She asked project participants to reflect and write about what it was like to work with the artists on these projects. In response to *Hiding*, members of the group thought the process made a space “between being and doing” and a “revolutionary” and “emergent” means to “explore (literally and figuratively) environments in a new way” (Participants, *Hiding in Plain Sight* 2016). For another participant, Jennifer Jackson, the project also offered a way to “reconcile realities and utopias” through thinking about action, process and role:

*“Inevitably, there is a distance between utopian practice and real-life practice. In this project, we aimed to inhabit and explore that space, to understand the gap in a tangible way” (Jackson, 2016 Participant, *Hiding in Plain Sight* 2016).*

Another – Mavis Machirori – saw the project as connected to an historic, anthropological process of defamiliarisation to “make the familiar alien in order to understand systems, processes and structures around us” (Machirori, 2016 Participant, *Hiding in Plain Sight* 2016). In her conception, the indeterminate “space” of the work reflected on the ambiguous “felt” realities of moving between types of professional performance.

For his part, Schrag’s project was also covered in the booklet by way of his own written text which sat alongside one by Tim Owen Jones, a participating nurse. For the subsequent film output, an off-spin of the project was devised in the form of *The Song of the Compassionate Robots*. This composite text – developed out of discussion at *The Secret Society* – was edited by Schrag and Made into an animation by artist John Harmer. It provided an engaging and accessible translation, but not a representation of the work. One might view it as something of stoodge or stand-in by-product: one, funny and lightweight enough to conform to the demand to “share findings in an imaginative and accessible way” (Bull, 2018) but without “shedding light” on the elusive dark space of *The Secret Society*. Its’ production certainly demonstrated that the deep and more risky and serious material generated by *The Secret Society* could be spun in more than one kind of way. But one can view *The Secret Society* itself as an absence in this context – one that took a deliberate line of flight away from the institutionally presentable products.

Williams’ inclusion of long texts written by participants themselves sought to capture a more complicated and committed process than simply learning outcomes or demonstrating “engagement.” The booklet marked an attempt to capture these fluid processes and associated insights. It represented an alternative approach to the existing pedagogies already in place at the healthcare school. These taught medical students to appreciate The Arts and Humanities through discussion of set-texts, delivered as part of a teaching module. Such approaches are able provide pre-determined “learning outcomes against which to measure success” (Dunton and Williams, 2016).

Such bracing, pre-determined assessment criteria dictated whether pilot projects were “worthy of future development and resource” (Participants, *Are You Feeling Better?* 2016). Despite the best efforts of the Learning Manager to show appreciation for the alternative forms of pedagogy offered by *Are you Feeling Better?* it was a test that, not altogether unsurprisingly, the programme failed to achieve. The final booklet sought to engineer some visibility so that the conversations could stick around long enough for the works to have some affect outside the communities that created them. While the capacity to create a kind of visibility might be yearned for, it’s also important to note that these projects that centre on experience and non-availability might also be construed as forms of cultural elitism, as Alex Farquarson notes.

*If “new institutionalism” cannot create these publics, it will remain an ambitious prototype, as hermetically sealed as the white cube it shrugs off (Farquharson, 2006).*

The project was not deeply embedded enough in the institution for it to be recognised as part of KCL’s data or outputs. Any wider production around potential publics ran into the buffers of diminishing resource, budgetary and human exhaustion alike. The publication, *Are You Feeling Better?* did not find a platform on the KCL website. But a later publication produced the following year similarly documented Arts in Health projects commissioned through the Cultural Institute (Arts in Mind King’s College London [KCL], 2018). Although this festival drew on the similar intention as *Are You Feeling Better?* – i.e., the engagement of more healthcare students in the arts – the artist projects developed this time around were more emphatically used to prop-up the edifice of the organisations’ narration of its own “success.” A prominently placed quote from one participant has been placed at the front of the report. Described as “audience feedback” (Arts in Mind King’s College London [KCL], 2018), it highlights, perhaps, the collapse of art education into forms of advertisement, advocacy and performance:

*I am a fifth year medic interested in psychiatry and neuroscience. Hearing about the psychiatric topics from all these different perspectives helps me set goals regarding the ideal standards I want to achieve in my career (Arts in Mind King’s College London [KCL], 2018).*

This awkward attempt to make audiences and types of publics both within the University and outside seems to be a particularly prevalent malady. Alan Read (et al), drawing on Bill Readings’ *The University in Ruins* and Maurice Merleau-Ponty’s lecture series *Institution and Passivity* delivers a damning analysis of the failure of education. To do this he explores what it means to “institute”: “a process of social formation, *a temporarily protracted development to endow experience with durable dimensions*” (Read et al., 2015). He explores how the University was instituted as a form for the nation-state, expressing an ideology of a shared community of difference. Most relevant to both artworks mentioned above, Read asserts the significance of dissonance as a process that resists easy institutionalisation, at the same time as operating to “increase the efficacy of the instituting process.” Schrag and Shaw’s work both performs this same uneasy instituting – making a new one, resisting the existing one, but at the same time recognising the value of dissonance, or agonism to better the existing one.

## Conclusion

*Are you Feeling Better?* and the two performative works explored here were born in an entangled (we might say *enstrangled*) landscape of desires and agendas that vie for centre stage: engagement, education, art and science and art and health. This mess is not novel or unusual but is perhaps just one rather complicated example, plucked from a familiar environment for contemporary commissioning in the United Kingdom. In developing art and health projects it is usual to iron away this complexity, conjuring into life clear and “perimetered” understandings of what is art, what is healthcare and what space constitutes the site for/of art.

The two projects attempt to live in this over-heated sea, but also to make the pressures part of the logic of the work. At the same time though both seek to make forms of “space” that offer a portal to another space, a parallel world with a slightly different climate. These spaces were different, wider, than the limited physical “space” of the show (where the work goes), the space of interaction (where engagement takes place) and affective space (the intended outcomes) that had been allocated by the commissioning context. Hybrid mutations of the actual institution were constructed, out of which new settings for performance became possible.

The works’ refusal to perform in the designated space allowed them, we would argue, to over-reach the “surface” ambition set for their limited success. Schrag’s *Secret Society of Imperfect Nurses* went underground, inhabiting the shadows. Perhaps it formed a parallel world, fed by feelings of personal failure, created by the relentless rhetoric of success patrolled by the institution. Likewise, Shaw’s work *Hiding in Plain Sight* literally burrows down into the material form of the institution, finding a type of escape from visibility on the surface.

It is interesting then, to return to the works and consider how they relate to an arts and health agenda that seeks different kinds of space. Arts and Health practices do not take place within some weightless, abstract nowhere, but are shaped by heavily weighed histories and sets of agendas that are performed within particular social and political contexts. Many of these institutions present complicated backdrops, stages and directions, alongside all-too-contemporary financial pressures demanding efficient uses of time, space and resource. Such combined forces strangle many Arts in Health project at birth or squeeze spaces of activity so narrowly that only the pre-conceived, the censored, the literal and the over-rehearsed can eek through. This institutional frame is rarely appraised or acknowledged in Arts in Health research.

Challenges to this disciplinary logic are now mounting and come from many corners of these intersecting disciplines. One comes from academics working with the Medical Humanities who have written on the (fraught) experience of working in creative partnerships as part of the current vogue for the inter-disciplinary. This is a concept which they comprehensively explore and critique in their work (Callard and Fitzgerald, 2016). They suggest that:

*one might approach a healthcare “institution” not as a conceptual and physical edifice whose histories we have become used to tracing (the National Health Service, the World Health Organisation, the hospital), but as something that gives form or order precisely by “cutting” or “disentangling” entities from a heterogeneous field (Callard and Fitzgerald, 2016: 42).*

The two projects we created, creatively engaged with some of the enstranglements and structural apparatus within which we had to find a place for the work (and ourselves). Those leading the charge for Critical Medical Humanities propose their own entangled field as one that has been constituted through forms of “intra-action” rather than “inter-action” (Barad, 2003). Using a provocative metaphor of cultural exchange, as a financial transaction, they assert that:

*We do not, as scholars from various disciplines, bring our objects and practices to another through a kind of free-trade agreement; rather we re-enter a long history of binding, tangling and cutting, within which current moves towards integration are much more weighted than they might at first seem (Callard and Fitzgerald, 2016: 39. *Emphasis added*).*

Such descriptions of the complex processes whereby exchanges of value and knowledge take place, rightly undermine the easy assertions, made by leaders at KCL, that the institution has long supported research into the “symbiosis between arts and health” (Utopia 2016 website, 2016) as though these separate entities are somehow self-evident and fixed, and not created through exactly the kind of thick, dense, accumulated processes and layerings of power, described above.

Critical reappraisals of Arts-Health practices and their conditions of production might further sit within broader accounts of the “health of critique” more broadly (Fassin, 2019). In a paper which asks the question, “How is critique?”, he playfully asks the reader to indulge him in giving a “medical bulletin,” if obviously one given “with a grain of salt.” (Spoiler: the news is not good!).

His main point is that “it is indispensable to contextualise critique both temporally and spatially,” making the point that “we must count for both dimensions” (Fassin, 2019: 14). He identifies how over the last half a century critique “has lost much of its radical edge and academic legitimacy, while being increasingly confined to marginal circles,” charting various contexts and places within which particular discourses rose and fell, (not least that of the “backlash” that began in the late 1970s when “the repressive neoliberal turn” began to take hold in the United States and United Kingdom). Thus, he concludes:

*The way in which research programs and scholars are currently promoted, funded, and assessed in institutions of higher education is a recent importation from the corporate world that has substantial effects on the production of knowledge. Not only can critique not ignore the structuring and interconnecting of these social spaces, but it is entirely embedded and shaped by them, even when it criticises them (Fassin, 2019: 21).*

While this may not be the place to further expand on the place and times of critique within which the formations of Arts-Health have sat (and sit), we believe the examples of practice detailed above point to productive points of specific constraint - pressures strongly informed by their time and place. As such, these specific examples bring wider applicable lessons in terms of how they relate to broader trends, directions and political economies that make visible “Arts-Health” as a useful category of thought and action, practice and research.

To conclude then, the opportunity to retrospectively write about these two projects in this journal has come as a welcome opportunity to revisit the site of a disappearance whose lessons have clung to those involved (if not the host body). Without the “space” in this journal to develop these afterthoughts, the trails described above might have gone entirely cold. The role of KCL in the field of Arts in Health, also continues to evolve – and expand – with many re-brandings and internal re-structurings made over the last 5 years, positioning an “arts enhanced health education” alongside a dedicated *Arts, Health and Well-being Hub* charged with “raising the university’s profile in this area, as leader, convenor, partner and participant.” As part of fresh conversations around the performance of Arts in Health in higher education, we hope the projects we describe here can challenge or spook future possibilities, suggest alternative forms of critical appraisals, and provide timely hauntings from lost pasts (7671).

## Ethics Statement

Written informed consent was obtained from the individual(s) for the publication of any potentially identifiable images or data included in this article.

## Author Contributions

All authors listed have made a substantial, direct, and intellectual contribution to the work, and approved it for publication.

## Conflict of Interest

The authors declare that the research was conducted in the absence of any commercial or financial relationships that could be construed as a potential conflict of interest.

## Publisher’s Note

All claims expressed in this article are solely those of the authors and do not necessarily represent those of their affiliated organizations, or those of the publisher, the editors and the reviewers. Any product that may be evaluated in this article, or claim that may be made by its manufacturer, is not guaranteed or endorsed by the publisher.

## Footnotes

1 Two of four commissions in total.
